# Supplementary material for: Association between statin use and risk of gallstone disease and cholecystectomy: a meta-analysis of 590,086 patients
Source: PeerJ. 2023 Apr 7;11:e15149. doi: 10.7717/peerj.15149 (PMC10084820; doi:10.7717/peerj.15149)
Supplement: Supplemental Information 1 — MOOSE checklist, Search Strategy, and Data extraction and data synthesis [file peerj-11-15149-s001.docx]

**Supplementary file**

**Supplemental Method 1.** Preferred Reporting Items for Systematic Reviews and Meta-Analyses (PRISMA) 2009 checklist

**Supplemental Method 2.** Meta-analysis Of Observational Studies in Epidemiology (MOOSE) checklist

**Supplemental Method 3.** Search strategy

**Supplemental Method 4.** Data extraction and data synthesis

**Supplemental Table 1.** Patient characteristics of included case-control studies

**Supplemental Table 2.** Patient characteristics of retrospective cohort studies

**Supplemental Table 3.** Studies reporting different duration of statin use (current users)

**Supplemental Table 4.** Studies reporting different duration of statin use (former users)

**Supplemental Table 5.** Quality assessment of included studies using the National Heart, Lung, and Blood Institute tool for case-control studies

**Supplemental Table 6.** Risk Of Bias In Non-randomized Studies- of Intervention (ROBINS-I) for retrospective cohort study

**Supplemental Method 1.** PRISMA 2009 checklist

| **Section/topic** | **#** | **Checklist item** | **Reported on page #** |
| --- | --- | --- | --- |
| **TITLE** | | |  |
| Title | 1 | Identify the report as a systematic review, meta-analysis, or both. | 1 |
| **ABSTRACT** | | |  |
| Structured summary | 2 | Provide a structured summary including, as applicable: background; objectives; data sources; study eligibility criteria, participants, and interventions; study appraisal and synthesis methods; results; limitations; conclusions and implications of key findings; systematic review registration number. | 3 |
| **INTRODUCTION** | | |  |
| Rationale | 3 | Describe the rationale for the review in the context of what is already known. | 4, 5 |
| Objectives | 4 | Provide an explicit statement of questions being addressed with reference to participants, interventions, comparisons, outcomes, and study design (PICOS). | 4, 5 |
| **METHODS** | | |  |
| Protocol and registration | 5 | Indicate if a review protocol exists, if and where it can be accessed (e.g., Web address), and, if available, provide registration information including registration number. | 5 |
| Eligibility criteria | 6 | Specify study characteristics (e.g., PICOS, length of follow-up) and report characteristics (e.g., years considered, language, publication status) used as criteria for eligibility, giving rationale. | 5 |
| Information sources | 7 | Describe all information sources (e.g., databases with dates of coverage, contact with study authors to identify additional studies) in the search and date last searched. | 5 |
| Search | 8 | Present full electronic search strategy for at least one database, including any limits used, such that it could be repeated. | 5  eMethod 3 |
| Study selection | 9 | State the process for selecting studies (i.e., screening, eligibility, included in systematic review, and, if applicable, included in the meta-analysis). | 5 |
| Data collection process | 10 | Describe method of data extraction from reports (e.g., piloted forms, independently, in duplicate) and any processes for obtaining and confirming data from investigators. | 5, 6  eMethod 4 |
| Data items | 11 | List and define all variables for which data were sought (e.g., PICOS, funding sources) and any assumptions and simplifications made. | 5, 6  eMethod 4 |
| Risk of bias in individual studies | 12 | Describe methods used for assessing risk of bias of individual studies (including specification of whether this was done at the study or outcome level), and how this information is to be used in any data synthesis. | 6 |
| Summary measures | 13 | State the principal summary measures (e.g., risk ratio, difference in means). | 6 |
| Synthesis of results | 14 | Describe the methods of handling data and combining results of studies, if done, including measures of consistency (e.g., I^2^) for each meta-analysis. | 6 |
| Risk of bias across studies | 15 | Specify any assessment of risk of bias that may affect the cumulative evidence (e.g., publication bias, selective reporting within studies). | 7 |
| Additional analyses | 16 | Describe methods of additional analyses (e.g., sensitivity or subgroup analyses, meta-regression), if done, indicating which were pre-specified. | 6, 7 |
| **RESULTS** |  |  |  |
| Study selection | 17 | Give numbers of studies screened, assessed for eligibility, and included in the review, with reasons for exclusions at each stage, ideally with a flow diagram. | 7  Figure 1 |
| Study characteristics | 18 | For each study, present characteristics for which data were extracted (e.g., study size, PICOS, follow-up period) and provide the citations. | 7  Table 1 |
| Risk of bias within studies | 19 | Present data on risk of bias of each study and, if available, any outcome level assessment (see item 12). | 8  eTable 1, |
| Results of individual studies | 20 | For all outcomes considered (benefits or harms), present, for each study: (a) simple summary data for each intervention group (b) effect estimates and confidence intervals, ideally with a forest plot. | 8  Figure 2 eFigure 1 |
| Synthesis of results | 21 | Present results of each meta-analysis done, including confidence intervals and measures of consistency. | 8  Figure 2 eFigure 1 |
| Risk of bias across studies | 22 | Present results of any assessment of risk of bias across studies (see Item 15). | 9  eFigure 3A, 3B |
| Additional analysis | 23 | Give results of additional analyses, if done (e.g., sensitivity or subgroup analyses, meta-regression (see Item 16). | 10, 11  Figure 3-5 eFigure 2 |
| **DISCUSSION** |  |  |  |
| Summary of evidence | 24 | Summarize the main findings including the strength of evidence for each main outcome; consider their relevance to key groups (e.g., healthcare providers, users, and policy makers). | 9 |
| Limitations | 25 | Discuss limitations at study and outcome level (e.g., risk of bias), and at review-level (e.g., incomplete retrieval of identified research, reporting bias). | 12 |
| Conclusions | 26 | Provide a general interpretation of the results in the context of other evidence, and implications for future research. | 13 |
| **FUNDING** |  |  |  |
| Funding | 27 | Describe sources of funding for the systematic review and other support (e.g., supply of data); role of funders for the systematic review. | 2 |

**Supplemental Method 2.** MOOSE checklist

| Reporting Criteria | Reported (Yes/No) | Reported on Page No. | Brief description |
| --- | --- | --- | --- |
| Reporting of Background |  |  |  |
| Problem definition | Yes | 4, 5 | To date, the protective effect of statin on gallstone disease is not well clarified. Previous meta-analysis had a considerable level heterogeneity remained unexplored. |
| Hypothesis statement | Yes | 4, 5 | Duration of statin use and discontinuation may affect the clinical outcomes |
| Description of Study Outcome(s) | Yes | 5, 6 | Risk of incident cholelithiasis and cholecystectomy |
| Type of exposure or intervention used | Yes | 5 | Statin users and non-users |
| Type of study design used | Yes | 5 | Randomized controlled trials, prospective studies, retrospective studies and case-control studies were included |
| Study population | Yes | 5 | Adults without previously diagnosed gallstone disease |
| Reporting of Search Strategy |  |  |  |
| Qualifications of searchers (eg, librarians and investigators) | Yes | 5 | Yu Chang and Kuan-Yu Chi |
| Search strategy, including time period included in the synthesis and keywords | Yes | 5, eMethod 3 | See eMethod 3 |
| Effort to include all available studies, including contact with authors | Yes | eMethod 3 | It is unnecessary to contact authors as the data were publicly available. |
| Databases and registries searched | Yes | 5, eMethod 3 | Embase, Medline and Cochrane library |
| Search software used, name and version, including special features used (eg, explosion) | Yes | 5, eMethod 3 | Endnote X 9.3 was used to manage reference |
| Use of hand searching (eg, reference lists of obtained articles) | Yes | 5, eMethod 3 | We searched bibliographies of retrieved references. |
| List of citations located and those excluded, including justification | Yes | Figure 1 | Details of the literature search process are outlined in the PRISMA flow diagram. The citation list for excluded studies is available upon request. |
| Method for addressing articles published in languages other than English | Yes | 5 | We placed no restrictions on language. |
| Method of handling abstracts and unpublished studies | Yes | 5 | We did not include any conference abstract. |
| Description of any contact with authors | Yes | eMethod 3 | It is unnecessary to contact authors as the data were publicly available. |
| Reporting of Methods |  |  |  |
| Description of relevance or appropriateness of studies assembled for assessing the hypothesis to be tested | Yes | 5 | (1) Randomized controlled trials, prospective studies, retrospective studies and case-control studies were included  (2) studies involving human adults without history of gallstone or cholecystectomy as participants  (3) studies reporting clinical outcome as with/without diagnosis of gallstone or record of cholecystectomy |
| Rationale for the selection and coding of data (eg, sound clinical principles or convenience) | Yes | 5, eMethod 4 | Two investigators (Y.C and K.Y.C) independently extracted relevant information from eligible articles. |
| Documentation of how data were classified and coded (eg, multiple raters, blinding, and interrater reliability) | Yes | eMethod 4 | Details of extraction are in eMethod 4. |
| Assessment of confounding (eg, comparability of cases and controls in studies where appropriate | Yes | 6 | Two reviewers (Y.C and H.M.L) independently completed a critical appraisal of included literature by using the Risk Of Bias In Non-randomized Studies - of Interventions (ROBINS-I) tool for each clinical outcome |
| Reporting Criteria |  |  |  |
| Assessment of study quality, including blinding of quality assessors; stratification or regression on possible predictors of study results | Yes | 6 | ROBINS-I tool |
| Assessment of heterogeneity | Yes | 6 | Heterogeneity was assessed using I^2^ statistics proposed by Higgins and Thompson. |
| Description of statistical methods (eg, complete description of fixed or random effects models, justification of whether the chosen models account for predictors of study results, dose-response models, or cumulative meta-analysis) in sufficient detail to be replicated | Yes | 6 | We performed meta-analysis using random-effects model  RStudio’s ‘‘metafor’’package was used for all analyses. |
| Provision of appropriate tables and graphics | Yes | Figure and Table | We included tables for illustrating details of included the studies and figures demonstrating a flow chart of study identification and the results of the meta-analyses. |
| Reporting of Results |  |  |  |
| Table giving descriptive information for each study included | Yes | 9 | Details are in Table 1 |
| Results of sensitivity testing (eg, subgroup analysis) | Yes | 7,8 | We performed subgroup analysis based on definition of gallstone disease, duration of statin use and different types of study. |
| Indication of statistical uncertainty of findings | Yes | 7,8 | 95% confidence intervals and I^2^ values were presented with all effect estimates |
| Reporting of Discussion |  |  |  |
| Quantitative assessment of bias (eg, publication bias) | Yes | 9, eFigure3A, 3B | See eFigure3A, 3B |
| Justification for exclusion (eg, exclusion of non–English-language citations) | Yes | 6 | Studies were excluded based on the pre-specified eligibility criteria in Method. |
| Assessment of quality of included studies | Yes | 8, eTable 1 | See eTable 1 |
| Reporting of Conclusions |  |  |  |
| Consideration of alternative explanations for observed results | Yes | 8 | Short-term use of statin may lead to an even higher risk of developing gallstones in women. Short-term use of statin may be associated with developing diabetes mellitus, which is also a risk factor of gallstone development since the formation of cholesterol gallstone is also a metabolic problem. |
| Generalization of the conclusions (ie, appropriate for the data presented and within the domain of the literature review) | Yes | 13 | The use of statins lowers the risk of gallstone disease, especially medium and long-term use. The protective effects of statins on gallstone disease was more pronounced in men than women |
| Guidelines for future research | Yes | 13 | We propose that prospective large multicenter studies should be undertaken to overcome the limitations. |
| Disclosure of funding source | Yes | 2 | Not applicable |

**Supplemental Method 3.** **Search strategy**

We searched bibliographies of retrieved references.

It is unnecessary to contact authors as the data were publicly available.

***EMBASE****:*

| Search Number | Search Description | Numbers of results |
| --- | --- | --- |
| 1 | ((Hydroxymethylglutaryl* adj5 inhibitor*) or (hmg coa* adj5 inhibit*) or "reductase inhibitor*" or ((cholesterol*or lipid*) adj3 lower* adj3 (drug*or agent*)) or simvastatin or fluvastatin or cerivastatin or lovastatin or pravastatin or atorvastatin or rosuvastatin or lipostat or lipitor or crestor or zocor or pravachol or baycol or lescol or mevacor or mevinolin ):ti,ab,kw,de | 180621 |
| 2 | 'hydroxymethylglutaryl coenzyme a reductase inhibitor'/exp | 177992 |
| 3 | ((gallstone*:ti,ab,kw,de OR gallbladder:ti,ab,kw,de) AND stone*:ti,ab,kw,de OR biliary:ti,ab,kw,de) AND stone*:ti,ab,kw,de OR cholelithiasis:ti,ab,kw,de OR cholecystectomy:ti,ab,kw,de OR cholecystitis:ti,ab,kw,de | 120058 |
| 4 | 'cholecystectomy'/exp OR 'cholelithiasis'/exp | 105698 |
| 5 | (1 OR 2) AND (3 OR 4) and [embase]/lim | 965 |

***Medline***

| Search Number | Search Description | Numbers of results |
| --- | --- | --- |
| 1 | ((Hydroxymethylglutaryl* adj5 inhibitor*) or (hmg coa* adj5 inhibit*) or "reductase inhibitor*" or (cholesterol*or lipid* adj3 lower* adj3 drug*or agent*) or simvastatin or fluvastatin or cerivastatin or lovastatin or pravastatin or atorvastatin or rosuvastatin or lipostat or lipitor or crestor or zocor or pravachol or baycol or lescol or mevacor or mevinolin).mp. [mp=title, abstract, original title, name of substance word, subject heading word, floating sub-heading word, keyword heading word, organism supplementary concept word, protocol supplementary concept word, rare disease supplementary concept word, unique identifier, synonyms | 59799 |
| 2 | exp " Hydroxymethylglutaryl-CoA Reductase Inhibitors"/ | 45142 |
| 3 | (gallstone* or gallbladder stone* or biliary stone* or cholelithiasis or cholecystectomy or cholecystitis).mp. [mp=title, abstract, original title, name of substance word, subject heading word, floating sub-heading word, keyword heading word, organism supplementary concept word, protocol supplementary concept word, rare disease supplementary concept word, unique identifier, synonyms] | 80851 |
| 4 | exp "cholelithiasis"/ or exp "cholecystectomy"/ | 57058 |
| 5 | (1 OR 2) AND (3 OR 4) | 134 |

***Cochrane***

| Search Number | Search Description | Numbers of results |
| --- | --- | --- |
| 1 | ((Hydroxymethylglutaryl* adj5 inhibitor*) or (hmg coa* adj5 inhibit*) or "reductase inhibitor*" or ((cholesterol*or lipid*) adj3 lower* adj3 (drug*or agent*)) or simvastatin or fluvastatin or cerivastatin or lovastatin or pravastatin or atorvastatin or rosuvastatin or lipostat or lipitor or crestor or zocor or pravachol or baycol or lescol or mevacor or mevinolin ) | 14698 |
| 2 | MeSH descriptor: [Hydroxymethylglutaryl-CoA Reductase Inhibitors] explode all trees | 3716 |
| 3 | (gallstone* or gallbladder stone* or biliary stone* or cholelithiasis or cholecystectomy or cholecystitis) | 8919 |
| 4 | MeSH descriptor: [Cholecystectomy] explode all trees | 2046 |
| 5 | MeSH descriptor: [Cholelithiasis] explode all trees | 1137 |
| 6 | (1 OR 2) AND (3 OR 4 OR 5), in Trials | 64 |

**Supplemental Method 4.** **Data extraction and data synthesis**

Two investigators (Y.C and K.Y.C) independently extracted relevant information from tables or results of eligible articles. Extracted data included first author name, publication year, country where the study was conducted, data resource, follow-up duration, number of participants, sex, age range, body mass index, underlying cardiovascular diseases, and method used to identify cases. Additionally, we obtained data about adjusted odds ratio (OR) and standard error (SE) from each study to collectively estimate the risk of gallstone in statin users. For studies that reported outcomes by relative risk (RR), since the extracted RR and SE were adjusted, the formula^3^ for the conversion between RR and OR maybe inappropriate. Therefore, we regarded the adjusted RR as OR since most of the included studies have incidence of outcome less than 10% and all ORs converted from RRs were between 0.5 and 2.5, correction may not be desirable^4^.

We used Rstudio with metafor packages to conduct statistical analysis

**【metafor】package**
Random-effects model:
Generic inverse variance meta-analysis (metagen)

Several plots for meta-analysis: Forest plot (forest)
Funnel plot (funnel)

Exploring biases:
Egger’s test (metabias)

**【metafor】packages**

Risk of gallstone disease:

gallstone.or <- metagen(logOR, selogOR, studlab=studyname, sm="OR", data=gallstone, method.tau='REML)

forest(gallstone.or, layout="RevMan5", lab.e="Statin user", lab.c=" Non-users ", xlab="Favors statin user Favors non-users", ff.xlab="bold", col.by="black", pooled.events=F, comb.random=T, comb.fixed=F, col.diamond.random=("red"), col.diamond.lines.random="red")

funnel(gallstone.or, comb.random = F, contour.levels = c(0.9,0.95,0.99), col.contour = c("dark blue","blue","light blue"), ref = exp(gallstone.or$TE.fixed))

> legend("topright", c("p < 0.01", "0.01 < p < 0.05", "0.05 < p < 0.10", "p > 0.10"), fill=c("light blue","blue","dark blue", 'white'), bg = 'white')

gallstone.bias <- metabias(gallstone.or,method.bias="linreg",plotit=T)

abline(h=c(0,-2,2), col = c("red","gray","gray"))

| **Supplemental Table 1. Patient characteristics of included case-control studies** | | | | | | | | | | | | | | | | | | | | | | |  |
| --- | --- | --- | --- | --- | --- | --- | --- | --- | --- | --- | --- | --- | --- | --- | --- | --- | --- | --- | --- | --- | --- | --- | --- |
| **Study, year** | **Sample size, n** | | | | | **Mean age at index (yrs)** | | **Female, n (%)** | | | | **Diabetes n (%)** | | | | **Hypertension, n (%)** | | | | **Hyperlipidemia, n (%)** | | |  |
|  | Case | Control | | Total | |  | | Case | | Control | | Case | | Control | | Case | | Control | | Case | | Control |  |
| Biétry 2016^23^ | 2220 | 8880 | | 11100 | | 61.8 | | 1315 (59.2) | | 5260 (59.2) | | 268 (12.1) | | 892 (10.1) | | N/A | | N/A | | N/A | | N/A |  |
| Bodmer 2009^19^ | 27035 | 106531 | | 133566 | | 53.4 | | 20656 (76.4) | | 81463 (76.5) | | N/A | | N/A | | N/A | | N/A | | N/A | | N/A |  |
| Chiu 2012^21^ | 1014 | 1014 | | 2028 | | 66.8 | | 456 (45.0) | | 456 (45.0) | | 195 (19.4) | | 203 (20.0) | | N/A | | N/A | | N/A | | N/A |  |
| Erichsen 2010^17^ | 32494 | 324925 | | 357419 | | 54.6 | | 22371 (68.9) | | 223702 (68.9) | | 1797 (5.5) | | 13183 (4.1) | | N/A | | N/A | | N/A | | N/A |  |
| González-Pérez 2007^18^ | 2353 | 10000 | | 12353 | | 53.4 | | 1704 (72.5) | | 7230 (72.3) | | 121 (5.1) | | 300 (3.0) | | 440 (18.7) | | 1503 (15.0) | | 277 (2.2) | | 4967 (1.5) |  |
| Merzon 2010^16^ | 1465 | 5860 | | 7325 | | 40-85 | | 972 (67) | | 3928 (67) | | 276 (19.0) | | 969 (16.5) | | N/A | | N/A | | 178 (7.6) | | 571 (5.7) |  |
| N/A, not applicable | |  | |  | |  | |  | |  | |  | |  | |  | |  | |  | |  |  |
| **Supplemental Table 1 (Continued)** | | | | | | | | | | | | | | | | | | | | | | |  |
| **Study,year** | **IHD, n (%)** | | | | **COPD, n (%)** | | | | **CVD, n (%)** | | | | **Cardiovascular disease, n (%)** | | | | **Cirrhosis, n (%)** | | | | **Thiazide, n (%)** | |  |
|  | Case | | Control | | Case | | Control | | Case | | Control | | Case | | Control | | Case | | Control | | Case | Control |  |
| Biétry 2016^23^ | 1221(55.0) | | 4093(46.1) | | N/A | | N/A | | 547 (29.1) | | 2118(23.9) | | N/A | | N/A | | N/A | | N/A | | N/A | N/A |  |
| Bodmer 2009^19^ | 2129(7.9) | | 11565(10.9) | | N/A | | N/A | | 611 (2.3) | | 2539 (2.4) | | N/A | | N/A | | N/A | | N/A | | N/A | N/A |  |
| Chiu 2012^21^ | 156 (15.4) | | 204(20.1) | | 212(20.91) | | 247(24.36) | | 182(18.0) | | 180(17.8) | | N/A | | N/A | | 34(3.4) | | 21(2.1) | | N/A | N/A |  |
| Erichsen 2010^17^ | N/A | | N/A | | 1487(4.6) | | 9807(3.0) | | 1049(3.2) | | 8531(2.6) | | 5387(16.6) | | 37737(11.6) | | 109(0.3) | | 488(0.2) | | 270(0.8) | 2085(0.6) |  |
| González-Pérez 2007^18^ | 277(11.8) | | 609(6.1) | | N/A | | N/A | | 109 (4.6) | | 263 (2.6) | | N/A | | N/A | | N/A | | N/A | | 189 (8) | 563 (5.6) |  |
| Merzon 2010^16^ | 221 (15.2) | | 966 (16.4) | | N/A | | N/A | | N/A | | N/A | | N/A | | N/A | | 14 (0.9) | | 13 (0.2) | | N/A | N/A |  |
| COPD, Chronic obstructive pulmonary disease, CVD, cerebrovascular disease, IHD, ischemic heart disease, N/A, not applicable | | | | | | | | | | | | | | | | | | | | | | |  |

| **Supplemental Table 1 (Continued)** | | | | | | | | | | | | | |
| --- | --- | --- | --- | --- | --- | --- | --- | --- | --- | --- | --- | --- | --- |
| **Study, year** | **Opposed estrogen 1-9,**  **n (%)** | | **Opposed estrogen ≥10,**  **n (%)** | | **Unopposed estrogen 1-9,**  **n (%)** | | **Unopposed estrogen ≥10,**  **n (%)** | | **HRT, n (%)** | | **Current use of statin, n (%)** | | |
|  | Case | Control | Case | Control | Case | Control | Case | Control | Case | Control | Case, n (%) | Control, n (%) | AOR (95% CI) |
| Biétry 2016^23^ | 80 (3.6) | 109 (2.1) | 37 (1.7) | 99 (1.1) | 239 (10.8) | 878 (9.9) | 81 (3.7) | 228 (2.6) | N/A | N/A | 396 (17.8) | 1527 (17.2) | 0.85 (0.74 to 0.99) |
| Bodmer 2009^19^ | 555 (2.1) | 1728(1.6) | 909 (3.4) | 2445 (2.3) | 570 (2.1) | 1267(1.2) | 1245(4.6) | 2565(2.4) | N/A | N/A | 1832 (6.8) | 7342 (6.9) | 0.78 (0.73 to 0.83) |
| Chiu 2012^21^ | N/A | N/A | N/A | N/A | N/A | N/A | N/A | N/A | N/A | N/A | 250 (24.7) | 240 (23.7) | 1.14 (0.90 to 1.43) |
| Erichsen 2010^17^ | N/A | N/A | N/A | N/A | N/A | N/A | N/A | N/A | 3376(10.4) | 25284 (7.8) | 1764 (5.4) | 15580 (4.8) | 0.93 (0.87 to 0.98) |
| González-Pérez 2007^18^ | N/A | N/A | N/A | N/A | N/A | N/A | N/A | N/A | N/A | N/A | 31 (1.3) | 105 (1.1) | 0.63 (0.39 to 1.04) |
| Merzon 2010^16^ | N/A | N/A | N/A | N/A | N/A | N/A | N/A | N/A | N/A | N/A | 392 (26.7) | 1773 (30.2) | 0.73 (0.62 to 0.87) |
| AOR, adjusted odds ratio, CI, confidence interval, HRT, hormone replacement therapy, N/A, not applicable, numbers of estrogen indicate prescriptions prior to the index date | | | | | | | | | | | | | |

| **Supplemental Table 2.** **Patient characteristics of retrospective cohort studies** | | | | | | | | | | | | | | |
| --- | --- | --- | --- | --- | --- | --- | --- | --- | --- | --- | --- | --- | --- | --- |
| **Study, year** |  | | **Sample size, n** |  | **Mean age, (year)** | | **Female, n (%)** | | **Diabetes, n (%)** | | **Hypertension, n (%)** | | **COPD, n (%)** | |
|  | | Statin user | Non-user | Total | Statin user | Non-user | Statin user | Non-user | Statin user | Non-user | Statin user | Non-user | Statin user | Non-user |
| Martin 2015^24^ | | 6342 | 6342 | 12684 | 55 | 56 | 2924 (46.1) | 2856 (45.0) | 789(12.4) | 743(11.7) | 3707(58.5) | 3766 (59.4 | 742(11.7) | 742(11.6) |
| Tsai 2009^15^ | | 7996 | 45615 | 53611 | 65.8 | 65.9 | 7996 (100) | 45615 (100) | 872 (10.9) | 2281 (5.0 | N/A | N/A | N/A | N/A |
| COPD, chronic obstructive pulmonary disease, N/A, not applicable | | | | | | | | | | | | | | |

| **Supplemental Table 2.** **(Continued)** | | | | | | | | | | | | | | |
| --- | --- | --- | --- | --- | --- | --- | --- | --- | --- | --- | --- | --- | --- | --- |
| **Study, year** | **Stroke or CVD, n (%)** | | | **CAD, n (%)** | | **Thiazide, n (%)** | | **HRT, n (%)** | | **Oral contraceptive, n (%)** | | **Gallstone disease, n (%)** | | |
|  | | Statin user | Non-user | Statin user | Non-user | Statin user | Non-user | Statin user | Non-user | Statin user | Non-user | Statin user | Non-user | AOR (95% CI) |
| Martin 2015^24^ | | 125 (2.0) | 128 (2.0) | 314 (5.0) | 277 (4.4) | N/A | N/A | 935 (14.7)) | 984 (15.5) | 430 (0.7) | 29 (0.5) | 270 (4.3) | 314 (5.0) | 0.86 (0.73 to 1.02) |
| Tsai 2009^15^ | | N/A | N/A | N/A | N/A | 992 (12.4) | 3786 (8.3) | 4414 (55.2) | 24085 (52.8) | 4238 (5.0) | 23647 (51.9) | 167 (2.1) | 2412 (5.3) | 0.82 (0.70 to 0.96) |
| CVD, cerebrovascular disease, CAD, coronary artery disease, HRT, hormone replacement therapy, N/A, not applicable | | | | | | | | | | | | | | |

| **Supplemental Table 3. Studies reporting different duration of statin use (current users)** | | | | | | | | | |
| --- | --- | --- | --- | --- | --- | --- | --- | --- | --- |
| **Study, year** | **1-4 prescriptions**  **Overall, n (%)** | | | **1-4 prescriptions**  **Men, n (%)** | | | **1-4 prescriptions**  **Women, n (%)** | | |
|  | Case | Control | AOR (95% CI) | Case | Control | AOR (95% CI) | Case | Control | AOR (95% CI) |
| Biétry 2016^23^ | 63 (2.8) | 155 (1.7) | 1.34 (0.99 to 1.83) | 34 (1.5) | 77 (0.9) | 1.50 (0.98 to 2.32) | 29 (1.3) | 78 (0.9) | 1.20 (0.77 to 1.88) |
| Bodmer 2009^19^ | 277 (1.0) | 832 (0.8) | 1.10 (0.95 to 1.27 | 89(0.3) | 312 (0.3) | 0.99 (0.77 to 1.26) | 188(0.7) | 520(0.5) | 1.19(0.99 to 1.42) |
| Erichsen 2010^17^ | 464 (1.4) | 3443 (1.1) | 1.17 (1.06 to 1.30) | 185(0.6) | 1434 (0.4) | 1.06(0.90 to 1.25) | 279(0.9) | 2009(0.6) | 1.25(1.1 to 1.42) |
|  | **5-19 prescriptions**  **Overall, n (%)** | | | **5-19 prescriptions**  **Men, n (%)** | | | **5-19 prescriptions**  **Women, n (%)** | | |
|  | Case | Control | AOR (95% CI) | Case | Control | AOR (95% CI) | Case | Control | AOR, 95 CI |
| Biétry 2016^23^ | 234 (10.5) | 1005 (11.3) | 0.77 (0.65 to 0.92) | 124 (5.6) | 546 (6.1 | 0.78 (0.61 to 1.00) | 110 (5.0) | 459 (5.2) | 0.77 (0.61 to 0.99) |
| Bodmer 2009^19^ | 690 (2.6) | 2550 (2.4) | 0.8 (0.77 to 0.93) | 256 (1.0) | 959 (0.9) | 0.89 (0.76 to 1.03) | 434 (1.6) | 1591 (1.5) | 0.84 (0.75 to 0.94) |
| Erichsen 2010^17^ | 813 (2.5) | 7368 (2.3) | 0.89 (0.82 to 0.97) | 349 (1.1) | 3,188 (1.0) | 0.84 (74 to 0.95) | 464 (1.4) | 4,180 (1.3) | 0.93 (0.84 to 1.03) |
|  | **≥20 prescriptions**  **Overall, n (%)** | | | **≥20 prescriptions**  **Men, n (%)** | | | **≥20 prescriptions**  **Women, n (%)** | | |
|  | Case | Control | AOR (95% CI) | Case | Control | AOR (95% CI) | Case | Control | AOR, 95 CI |
| Biétry 2016^23^ | 99 (4.5) | 367 (4.1) | 0.88 (0.69 to 1.13) | 60 (2.7) | 211 (2.4) | 0.98 (0.70 to 1.37) | 39 (1.8) | 156 (1.8) | 0.79 (0.54 to 1.16) |
| Bodmer 2009^19^ | 865 (3.2) | 3960 (3.7) | 0.64 (0.59 to 0.70) | 377 (1.4) | 1636 (1.5) | 0.70 (0.61-0.81) | 488 (1.8) | 2324 (2.2) | 0.61 (0.55-0.69) |
| Erichsen 2010^17^ | 487 (1.5) | 4769 (1.5) | 0.76 (0.69 to 0.84) | 207 (0.6) | 2,111 (0.6) | 0.69 (0.59 to 0.81) | 280 (0.9) | 2,658 (0.8) | 0.81 (0.70 to 0.92) |
| 1-4 prescriptions means the short-term use of statin, 5-19, medium-term, over 20, long-term, AOR, adjusted odds ratio, CI, confidence interval | | | | | | | | | |

| **Supplemental Table 4. Studies reporting different duration of statin use (former users)** | | | | |
| --- | --- | --- | --- | --- |
| **Study, year** | **1-4 prescriptions, n (%)** | | |  |
|  | Case | Control | AOR (95% CI) |  |
| Biétry 2016^23^ | 95 (4.3) | 339 (3.8) | 0.98 (0.77 to 1.26) |  |
| Bodmer 2009^19^ | 269 (1.0) | 722 (0.7) | 1.22 (1.05 to 1.41) |  |
| Erichsen 2010^17^ | 353 (1.1) | 2408 (0.7) | 1.24 (1.11 to 1.39) |  |
|  | **5-19 prescriptions, n (%)** | | |  |
|  | Case | Control | AOR (95% CI) |  |
| Biétry 2016^23^ | 83 (3.7) | 284 (3.2) | 1.00 (0.77 to 1.30) |  |
| Bodmer 2009^19^ | 205 (0.8) | 534 (0.5) | 1.20 (1.01 to 1.42) |  |
| Erichsen 2010^17^ | 299 (0.9) | 2,441 (0.7) | 0.97 (0.86 to 1.10) |  |
|  | **≥20 prescriptions, n (%)** | | |  |
|  | Case | Control | AOR (95% CI) |  |
| Biétry 2016^23^ | 5 (0.2) | 17 (0.2) | 1.06 (0.39 to 2.88) |  |
| Bodmer 2009^19^ | 90 (0.3) | 270 (0.3) | 0.97 (0.75 to 1.25) |  |
| Erichsen 2010^17^ | 109 (0.3) | 1040 (0.3) | 0.79 (0.64 to 0.97) |  |
| 1-4 prescriptions means the short-term use of statin, 5-19, medium-term, over 20, long-term, AOR, adjusted odds ratio, CI, confidence interval | | | | |

**Supplemental Table 5. Quality assessment of included studies using the National Heart, Lung, and Blood Institute tool for case-control studies**

**Biétry 2016**

| **Criteria** | **Yes** | **No** | **Other** |
| --- | --- | --- | --- |
| 1.  Was the research question or objective in this paper clearly stated and appropriate? | X |  |  |
| 2. Was the study population clearly specified and defined? | X |  |  |
| 3.  Did the authors include a sample size justification? |  | X |  |
| 4. Were controls selected or recruited from the same or similar population that gave rise to the cases (including the same timeframe)? | X |  |  |
| 5.  Were the definitions, inclusion and exclusion criteria, algorithms or processes used to identify or select cases and controls valid, reliable, and implemented consistently across all study participants? | X |  |  |
| 6. Were the cases clearly defined and differentiated from controls? | X |  |  |
| 7. If less than 100 percent of eligible cases and/or controls were selected for the study, were the cases and/or controls randomly selected from those eligible? |  |  | CD |
| 8.  Was there use of concurrent controls? |  | X |  |
| 9. Were the investigators able to confirm that the exposure/risk occurred prior to the development of the condition or event that defined a participant as a case? | X |  |  |
| 10. Were the measures of exposure/risk clearly defined, valid, reliable, and implemented consistently (including the same time period) across all study participants? | X |  |  |
| 11. Were the assessors of exposure/risk blinded to the case or control status of participants? |  |  | NA |
| 12. Were key potential confounding variables measured and adjusted statistically in the analyses? If matching was used, did the investigators account for matching during study analysis? | X |  |  |
| *CD, cannot determine; NA, not applicable; NR, not reported | | | |
| Quality Rating: Fair | | | |

**Bodmer 2009**

| **Criteria** | **Yes** | **No** | **Other** |
| --- | --- | --- | --- |
| 1.  Was the research question or objective in this paper clearly stated and appropriate? | X |  |  |
| 2. Was the study population clearly specified and defined? | X |  |  |
| 3.  Did the authors include a sample size justification? | X |  |  |
| 4. Were controls selected or recruited from the same or similar population that gave rise to the cases (including the same timeframe)? | X |  |  |
| 5.  Were the definitions, inclusion and exclusion criteria, algorithms or processes used to identify or select cases and controls valid, reliable, and implemented consistently across all study participants? | X |  |  |
| 6. Were the cases clearly defined and differentiated from controls? | X |  |  |
| 7. If less than 100 percent of eligible cases and/or controls were selected for the study, were the cases and/or controls randomly selected from those eligible? | X |  |  |
| 8.  Was there use of concurrent controls? |  | X |  |
| 9. Were the investigators able to confirm that the exposure/risk occurred prior to the development of the condition or event that defined a participant as a case? | X |  |  |
| 10. Were the measures of exposure/risk clearly defined, valid, reliable, and implemented consistently (including the same time period) across all study participants? | X |  |  |
| 11. Were the assessors of exposure/risk blinded to the case or control status of participants? |  |  | NA |
| 12. Were key potential confounding variables measured and adjusted statistically in the analyses? If matching was used, did the investigators account for matching during study analysis? | X |  |  |
| *CD, cannot determine; NA, not applicable; NR, not reported | | | |
| Quality Rating: Good | | | |

**Chiu 2012**

| **Criteria** | **Yes** | **No** | **Other** |
| --- | --- | --- | --- |
| 1.  Was the research question or objective in this paper clearly stated and appropriate? | X |  |  |
| 2. Was the study population clearly specified and defined? | X |  |  |
| 3.  Did the authors include a sample size justification? |  | X |  |
| 4. Were controls selected or recruited from the same or similar population that gave rise to the cases (including the same timeframe)? | X |  |  |
| 5.  Were the definitions, inclusion and exclusion criteria, algorithms or processes used to identify or select cases and controls valid, reliable, and implemented consistently across all study participants? | X |  |  |
| 6. Were the cases clearly defined and differentiated from controls? | X |  |  |
| 7. If less than 100 percent of eligible cases and/or controls were selected for the study, were the cases and/or controls randomly selected from those eligible? |  |  | CD |
| 8.  Was there use of concurrent controls? |  | X |  |
| 9. Were the investigators able to confirm that the exposure/risk occurred prior to the development of the condition or event that defined a participant as a case? | X |  |  |
| 10. Were the measures of exposure/risk clearly defined, valid, reliable, and implemented consistently (including the same time period) across all study participants? | X |  |  |
| 11. Were the assessors of exposure/risk blinded to the case or control status of participants? |  |  | NA |
| 12. Were key potential confounding variables measured and adjusted statistically in the analyses? If matching was used, did the investigators account for matching during study analysis? | X |  |  |
| *CD, cannot determine; NA, not applicable; NR, not reported | | | |
| Quality Rating: Fair | | | |

**Erichsen 2010**

| **Criteria** | **Yes** | **No** | **Other** |
| --- | --- | --- | --- |
| 1.  Was the research question or objective in this paper clearly stated and appropriate? | X |  |  |
| 2. Was the study population clearly specified and defined? | X |  |  |
| 3.  Did the authors include a sample size justification? |  | X |  |
| 4. Were controls selected or recruited from the same or similar population that gave rise to the cases (including the same timeframe)? | X |  |  |
| 5.  Were the definitions, inclusion and exclusion criteria, algorithms or processes used to identify or select cases and controls valid, reliable, and implemented consistently across all study participants? | X |  |  |
| 6. Were the cases clearly defined and differentiated from controls? | X |  |  |
| 7. If less than 100 percent of eligible cases and/or controls were selected for the study, were the cases and/or controls randomly selected from those eligible? | X |  |  |
| 8.  Was there use of concurrent controls? |  | X |  |
| 9. Were the investigators able to confirm that the exposure/risk occurred prior to the development of the condition or event that defined a participant as a case? | X |  |  |
| 10. Were the measures of exposure/risk clearly defined, valid, reliable, and implemented consistently (including the same time period) across all study participants? | X |  |  |
| 11. Were the assessors of exposure/risk blinded to the case or control status of participants? |  |  | NA |
| 12. Were key potential confounding variables measured and adjusted statistically in the analyses? If matching was used, did the investigators account for matching during study analysis? | X |  |  |
| *CD, cannot determine; NA, not applicable; NR, not reported | | | |
| Quality Rating: Good | | | |

**Gonza´lez-Pe´rez 2007**

| **Criteria** | **Yes** | **No** | **Other** |
| --- | --- | --- | --- |
| 1.  Was the research question or objective in this paper clearly stated and appropriate? | X |  |  |
| 2. Was the study population clearly specified and defined? | X |  |  |
| 3.  Did the authors include a sample size justification? |  | X |  |
| 4. Were controls selected or recruited from the same or similar population that gave rise to the cases (including the same timeframe)? | X |  |  |
| 5.  Were the definitions, inclusion and exclusion criteria, algorithms or processes used to identify or select cases and controls valid, reliable, and implemented consistently across all study participants? | X |  |  |
| 6. Were the cases clearly defined and differentiated from controls? | X |  |  |
| 7. If less than 100 percent of eligible cases and/or controls were selected for the study, were the cases and/or controls randomly selected from those eligible? |  |  | CD |
| 8.  Was there use of concurrent controls? |  | X |  |
| 9. Were the investigators able to confirm that the exposure/risk occurred prior to the development of the condition or event that defined a participant as a case? | X |  |  |
| 10. Were the measures of exposure/risk clearly defined, valid, reliable, and implemented consistently (including the same time period) across all study participants? | X |  |  |
| 11. Were the assessors of exposure/risk blinded to the case or control status of participants? |  |  | NA |
| 12. Were key potential confounding variables measured and adjusted statistically in the analyses? If matching was used, did the investigators account for matching during study analysis? | X |  |  |
| *CD, cannot determine; NA, not applicable; NR, not reported | | | |
| Quality Rating: Fair | | | |

**Merzon 2010**

| **Criteria** | **Yes** | **No** | **Other** |
| --- | --- | --- | --- |
| 1.  Was the research question or objective in this paper clearly stated and appropriate? | X |  |  |
| 2. Was the study population clearly specified and defined? | X |  |  |
| 3.  Did the authors include a sample size justification? |  | X |  |
| 4. Were controls selected or recruited from the same or similar population that gave rise to the cases (including the same timeframe)? | X |  |  |
| 5.  Were the definitions, inclusion and exclusion criteria, algorithms or processes used to identify or select cases and controls valid, reliable, and implemented consistently across all study participants? | X |  |  |
| 6. Were the cases clearly defined and differentiated from controls? | X |  |  |
| 7. If less than 100 percent of eligible cases and/or controls were selected for the study, were the cases and/or controls randomly selected from those eligible? | X |  |  |
| 8.  Was there use of concurrent controls? |  | X |  |
| 9. Were the investigators able to confirm that the exposure/risk occurred prior to the development of the condition or event that defined a participant as a case? | X |  |  |
| 10. Were the measures of exposure/risk clearly defined, valid, reliable, and implemented consistently (including the same time period) across all study participants? | X |  |  |
| 11. Were the assessors of exposure/risk blinded to the case or control status of participants? |  |  | NA |
| 12. Were key potential confounding variables measured and adjusted statistically in the analyses? If matching was used, did the investigators account for matching during study analysis? | X |  |  |
| *CD, cannot determine; NA, not applicable; NR, not reported | | | |
| Quality Rating: Good | | | |

**Supplemental Table 6.** Risk Of Bias In Non-randomized Studies- of Intervention (ROBINS-I) for retrospective cohort study

| **Study/Bias** | **Confounding** | **Selection of participants** | **Classification of interventions** | **Deviation from intended interventions** | **Missing data** | **Measurement of outcomes** | **Selection of the reported result** | **Overall** |
| --- | --- | --- | --- | --- | --- | --- | --- | --- |
| **Martin 2015** | **Moderate** | **Low** | **Low** | **Low** | **Low** | **Low** | **Low** | **Moderate** |
| **Tsai 2009** | **Moderate** | **Low** | **Low** | **Low** | **Low** | **Low** | **Low** | **Moderate** |
